# Characterization of the complete chloroplast genome of *Chlorophytum comosum* (Liliaceae)

**DOI:** 10.1080/23802359.2020.1721362

**Published:** 2020-02-03

**Authors:** Yu-Jiao Peng, Xue-Yu Cui, Meng-Chao Tan, Lin Hu, Hong-Yan Ruan, Yuan-Yuan Shao, En-Liang Song, Yu-Juan Tang

**Affiliations:** a Key Laboratory of Beibu Gulf Environment Change and Resources Utilization of Ministry of Education, Nanning Normal University, Nanning, P.R. China;; b Guangxi Key Laboratory of Earth Surface Processes and Intelligent Simulation, Nanning Normal University, Nanning, P.R. China;; c Guangxi Geographical Indication Crops Research Center of Big Data Mining and Experimental Engineering Technology, Nanning Normal University, Nanning, P.R. China;; d Guangxi Subtropical Crops Research Institute, Nanning, P.R. China

**Keywords:** *Chlorophytum comosum*, complete chloroplast genome, Liliaceae, Illumina sequencing

## Abstract

*Chlorophytum comosum* is a perennial ornamental plant in the family Liliaceae, it is also a valuable medicinal plant. To enrich the genetic resources of *C. comosum*, its chloroplast genome was determined by Illumina sequencing data. The chloroplast genome is a typical quadripartite structure with a size of 153,983 bp, of which the LSC region is 83,471 bp, the SSC region is 18,010 bp, and the pair of IR regions is 26,251 bp. The overall GC content is 37%. It contains 131 genes, including 85 protein-coding genes, 38 tRNA genes, and 8 rRNA genes. Phylogenetic analyses showed that *C. comosum* is closely related to *Chlorophytum rhizopendulum*. However, it can be distinguished from other plants. This study enriches the sequence resources of *C. comosum* and provides important data for the development of molecular identification markers.

*Chlorophytum comosum* is a perennial evergreen herb of the Liliaceae family, native to the tropics and southern Africa, and then spread to other parts of the world (Sharma et al. 2019). *Chlorophytum comosum* is mainly cultivated for home decoration and landscaping. Interestingly, the root of this species is also used as a herbal medicine because it is thought to have a pharmacological effect (Kaushik 2005). The genus *Chlorophytum* has more than 215 species (Chu et al. 2018). However, morphological similarities make it difficult to distinguish some species within the genus. The chloroplast genome is stable in structure and the genetic composition is conservative, and it is widely used in the study of plant phylogeny. However, so far, genetic and genomic data on this species are quite limited. This paper reported the complete chloroplast genome sequence of *C. comosum*, aiming to enrich the plastid genome information for molecular identification.

The samples of *C. comosum* were collected from the flower market at 22 Yongwu Road, Chaoyang Street, Xixiangtang District, Nanning City, Guangxi province (108°20′43″E, 22°54′7″N), the voucher specimen was stored in the Key Laboratory of the Ministry of Education, Nanning Normal University (specimen code NN201908). Total genomic DNA was extracted from 100 mg fresh leaves using the modified CTAB method, DNA libraries with an average length of 350 bp were constructed using the NexteraXT DNA library preparation Kit. The DNA libraries were sequenced on the Illumina NovaSeq platform. The resulting Illumina raw sequence reads were edited using NGS QC Tool Kit v2.3.3 (Patel and Jain 2012), the obtained high-quality reads were *de novo* assembled using SPAdesv.3.11.0 (Bankevich et al. 2012), and finally annotated by Plann software (Huang and Cronk 2015). The complete chloroplast genome of *C. comosum* (GenBank accession no. MN871944) is 153,983 bp in size, including two inverted repeats (IRs, 26,251 bp each), a large single-copy region (LSC, 83,471 bp), and a small single-copy region (SSC, 18,010 bp). A total of 131 genes were annotated, containing 85 protein-coding genes, 38 tRNA genes, and 8 rRNA genes. In addition, the overall GC content is 37%.

To analyze the phylogeny of *C. comosum*, 11 chloroplast genome sequences were aligned by HomBlocks software (Bi et al. 2018), and then trimmed using the Gblock method. Finally, the maximum likelihood (ML) phylogenetic tree was constructed by RAxML v8.2.9 software with 1000 bootstrap replicates (Stamatakis 2014). The result of ML phylogenetic tree showed that the phylogenetic relationship of *C. comosum* is close to that of *C. rhizopendulum*, but distant to that of *Caltha palustris* (Figure 1). Our results provide useful genetic information for species identification and phylogenetic reconstruction of *C. comosum*.

**Figure 1.**
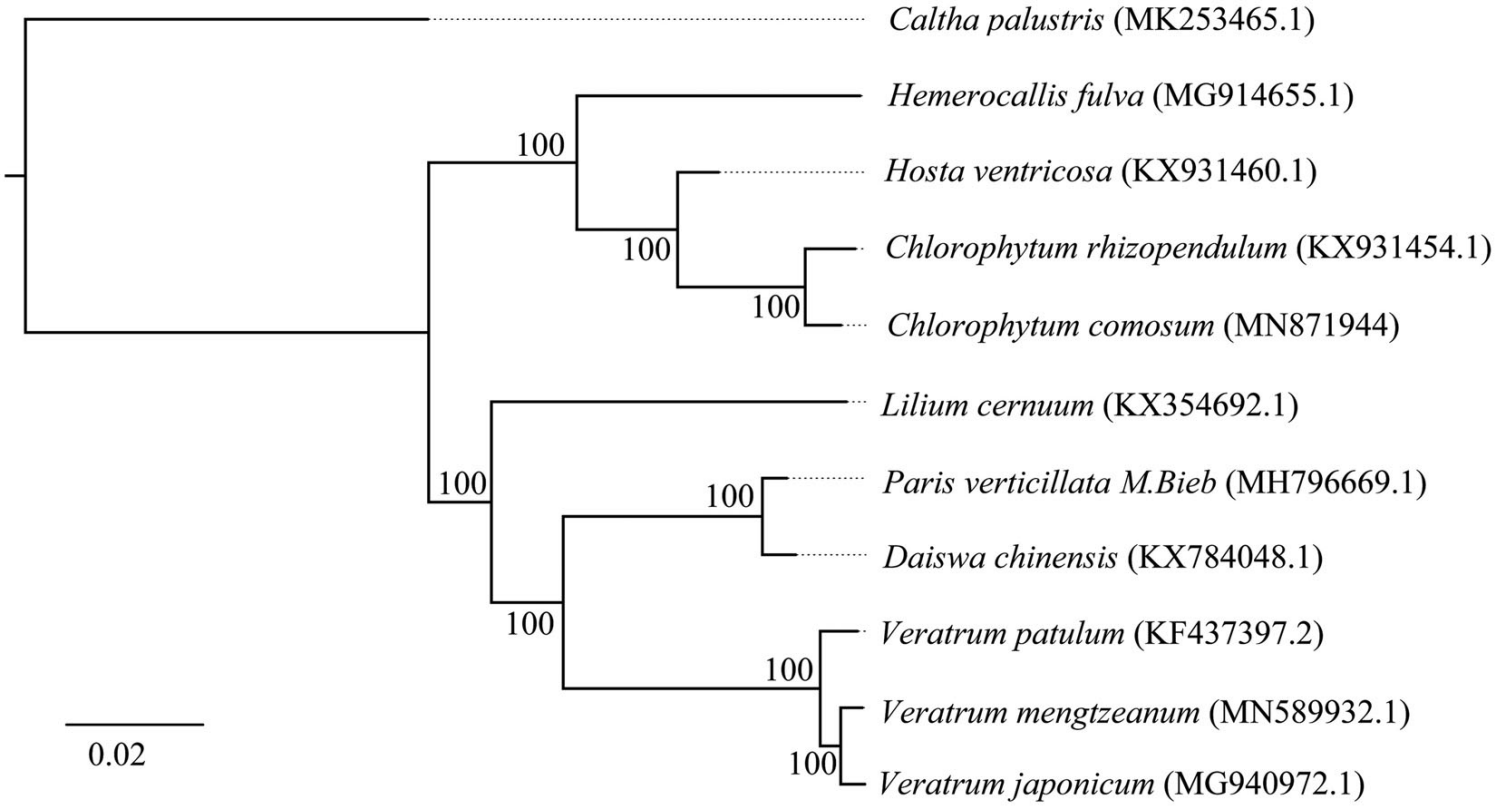
The maximum likelihood (ML) phylogenetic tree based on the chloroplast genomes of 11 species. Note: Numbers at the right of nodes represent the support value of 1000 bootstrap replicates.
